# Nonvalvular Atrial Fibrillation and Anticoagulation in the Elderly: A Retrospective Cohort Study

**DOI:** 10.14740/cr2219

**Published:** 2026-06-05

**Authors:** Kaiyu Jia, Elizabeth Rimsky, Joaquim Noguer, Aysan Sattarzadeh, Sabina Shah, Maryam Arooba Sherani, Omar Khayat, Bahy Abofrekha, Un Jung Lee, Suzanne El-Sayegh

**Affiliations:** a Department of Medicine, Northwell Health at Staten Island University Hospital, Staten Island, NY 10305, USA; b Department of Biostatistics, Northwell Health at Staten Island University Hospital, Staten Island, NY 10305, USA; c Department of Nephrology, Northwell Health at Staten Island University Hospital, Staten Island, NY 10305, USA

**Keywords:** Nonvalvular atrial fibrillation, Anticoagulation, Underutilization, Appropriateness, CHA_2_DS_2_-VASc, ORBIT

## Abstract

**Background:**

Atrial fibrillation (AF) is associated with a substantially increased risk of ischemic stroke and systemic embolism particularly in elderly patients. Understanding real-world patterns of anticoagulation (AC) use in elderly population is critical.

**Methods:**

This is a retrospective, single-center, chart review study that included patients who had nonvalvular AF between 2021 and 2024. Patients were divided into two groups according to age: 50–75 years old and 76–89 years old. Primary endpoint was AC underutilization, which was defined by the presence of all the following conditions: 1) CHA_2_DS_2_-VASc score > 2; 2) Outcomes Registry for Better Informed Treatment (ORBIT) score ≥ 3; 3) without AC. Secondary endpoint was AC appropriateness, which is defined by the presence of all the following conditions: 1) CHA_2_DS_2_-VASc > 2; 2) ORBIT < 3; 3) with AC.

**Results:**

A total of 6,386 patients were included. Among them, 3,306 patients were 50–75 years old, and 3,080 patients were 76–89 years old. Patients in age group 76–89 had a 4.547-fold higher odds ratio (OR = 4.547; 95% confidence interval (CI), 3.827–5.403) to have AC underutilization than those in age group 50–75. Patients in age group 76–89 were 108% (OR = 2.077; 95% CI, 1.818–2.374) more likely to have AC appropriateness than those in age group 50–75. History of bleeding (OR = 10.582; 95% CI, 6.467–17.315; P < 0.001) and glomerular filtration rate (GFR) (OR = 0.985; 95% CI, 0.982–0.988; P < 0.001) were independent predictors of appropriateness. Similar, history of bleeding (OR = 2.402; 95% CI, 1.666–3.462; P < 0.001) and GFR (OR = 1.006; 95% CI, 1.001–1.010; P = 0.0185) were also independent predictors of underutilization.

**Conclusions:**

Patients in age group 76–89 were more likely to have AC underutilization than patients in age group 50–75. History of bleeding and GFR were independent predictors of appropriateness and underutilization. These findings highlight the ongoing challenge of balancing stroke prevention and bleeding risk in elderly AF patients and underscore the importance of guideline-directed, individualized AC strategies.

## Introduction

Atrial fibrillation (AF) is associated with a substantially increased risk of ischemic stroke and systemic embolism particularly in elderly patients [1, 2]. Direct oral anticoagulants (DOACs) remain the cornerstone of stroke prevention in AF, with current guidelines recommending risk stratification using the CHA_2_DS_2_-VASc score to guide anticoagulation (AC) therapy [3, 4]. Although AC significantly reduces thromboembolic risk, its use in older adults is often complicated by concerns regarding bleeding. Elderly patient population is associated with both higher risks of stroke and bleeding [3, 4]. The Outcomes Registry for Better Informed Treatment (ORBIT) score provides risk stratification and may contribute to the decision-making in the initiation of AC [5].

Given the aging population and the rising prevalence of AF, understanding real-world patterns of AC use with consideration of various bleeding risks across patient populations of different ages is critical. In this retrospective study, we aim to investigate the prevalence of AC underutilization and appropriateness among patients with AF across different age groups. Our secondary objectives include exploring the difference of AC use within different age groups and the association of AC appropriateness with the incidence of stroke.

## Materials and Methods

### Study population

This is a retrospective, single-center, chart review study. Inclusion criteria were as follows: 1) patients who were 50 years and older; 2) patients who had nonvalvular AF (identified via International Classification of Diseases, 10th Revision (ICD-10) codes): 3) patients who had outpatient, inpatient or emergency department visits between 2021 and 2024 (including both AF and non-AF related reasons). Exclusion criteria were as follows: 1) patients who were < 50 years old; 2) patients without AF; 3) patients with mitral stenosis, aortic valve disease, or valve replacement surgery (i.e., valvular AF), identified through ICD-10 codes; 4) patients with ICD-10 codes indicating death documented prior to the diagnosis of AF; and 5) patients with missing AC data. Patients were divided into two groups according to age: 50–75 years old and 76–89 years old. Age stratification was performed to reflect clinically meaningful distinctions while maintaining balanced and adequate subgroup size for analysis and optimizing statistical power. Specifically, age 75 years was chosen because it represents an important threshold in AF management and risk stratification, including incorporation into the CHA_2_DS_2_-VASc score, where age ≥ 75 years carries increased weight due to its strong association with thromboembolic events.

### Endpoints definitions

Underutilization and appropriateness of AC use were defined according to a comprehensive assessment of stroke and bleeding risks according to the current guidelines [4, 6]. Given the complexity of the included patient cohort with multiple comorbidities and diverse clinical scenarios, the clinical decision of the included patients regarding AC management was made using an individualized approach, which takes into account various clinical factors and in adherence with current guidelines. However, in order to derive clinical patterns from the patient cohort rather than focusing purely on the individualized management of each patient, the CHA_2_DS_2_-VASc score and ORBIT score were used to standardize the assessment of AC underutilization and appropriateness, as they are able to serve as scalable tools or feasible algorithms to identify real-world actionable patterns, in which clinicians may weigh both thromboembolic risk and perceived bleeding risk when making AC decisions in elderly patients.

### Primary endpoint

The primary endpoint was the underutilization of AC therapy. Underutilization is defined by the presence of all the following conditions: 1) CHA_2_DS_2_-VASc > 2; 2) ORBIT ≥ 3; 3) without AC.

### Secondary endpoint

The secondary endpoint was the appropriateness of AC therapy. Appropriateness is defined by the presence of all the following conditions: 1) CHA_2_DS_2_-VASc > 2; 2) ORBIT < 3; 3) with any form of therapeutic AC (including DOACs, intravenous heparin, enoxaparin etc., not including deep vein thrombosis (DVT) prophylaxis).

Clinical outcomes such as ischemic stroke were identified via ICD-10 codes with documentation of the diagnosis time. The temporal markers of the stroke ICD codes were taken into account in the statistical analysis. If the documentation of ICD-10 codes occurred prior to that of AF, it was considered to be a comorbidity. *Vice versa*, it was considered as a clinical outcome in the univariate and multivariate regression analysis. “Active bleeding” refers to documentation of bleeding during the same indexed admission as the documentation of AF; “history of bleeding” refers to documentation of bleeding prior the same indexed admission as the documentation of AF; “bleeding” refers to the combination of “active bleeding” and “history of bleeding”. The ICD-10 codes regarding “active bleeding”, “history of bleeding”, and “bleeding” that were identified included only the clinically significant (or major) types of bleeding, such as gastrointestinal bleeding, intracranial hemorrhage, and hematuria.

### Statistical analysis

Statistical analysis was performed for the demographic and clinical characteristics of all populations and by age groups. Continuous variables were represented as median and interquartile range, and categorical variables were represented as count and percentage. All group comparisons were assessed using Wilcoxon rank sum test or Chi-square tests, as appropriate. Logistic regression was also performed to assess the prevalence and association of underutilization of AC therapy with age groups. Logistic regression was conducted to assess the association of the appropriateness of clinical decisions against AC with age group. In addition, Chi-square test was performed to explore the association of the appropriateness with incidence of ischemic stroke. All statistical tests were two-sided, with a significance level of 0.05. All analyses were conducted using SASV9.4 (SAS Institute, Cary, NC).

### Ethical compliance

This study was approved by the Institutional Review Board of our institution. All aspects of the study and patient care were conducted in strict adherence to the Hippocratic Oath and Health Insurance Portability and Accountability Act (HIPAA) regulations.

## Results

### Baseline characteristics

A total of 6,386 patients were included. Among them, 3,306 patients were 50–75 years old, and 3,080 patients were 76–89 years old. Baseline demographic and clinical characteristics were shown in Table 1. Compared to patients aged 50–75 years, those aged 76–89 years had higher rates of active bleeding (8.5% vs. 10.88%, P = 0.0013) and antiplatelet use (15.49% vs. 18.34%, P = 0.0023). They also had higher rates of comorbidities, such as systolic and/or diastolic heart failure (10.92% vs. 13.05%, P = 0.0087) and stroke (4.75% vs. 6.17%, P = 0.0124).

**Table 1:** Demographic and Clinical Characteristics

	Overall (n = 6,386)	Age 50–75 (n = 3,306)	Age 76–89 (n = 3,080)	P value
Age^a^	75 (67–81)	67 (62–72)	81 (79–85)	
CHA_2_DS_2_-VASc^a^ score	3 (2.0–4.0)	2 (1.0–3.0)	4 (3.0–4.0)	< 0.0001
GFR^a^	55 (34–76)	61 (37–82)	50 (32–68)	< 0.0001
ORBIT score^a^	2 (1.0–3.0)	1 (0–2)	2 (1.0–3.0)	< 0.0001
Active bleeding^b^				0.0013
No	5,770 (90.35%)	3,025 (91.5%)	2,745 (89.12%)	
Yes	616 (9.65%)	281 (8.5%)	335 (10.88%)	
Antiplatelet use^b^				0.0023
No	5,309 (83.13%)	2,794 (84.51%)	2,515 (81.66%)	
Yes	1,077 (16.87%)	512 (15.49%)	565 (18.34%)	
History of bleeding^b^				0.556
No	4,447 (69.64%)	2,313 (69.96%)	2,134 (69.29%)	
Yes	1,939 (30.36%)	993 (30.04%)	946 (30.71%)	
HFrEF or HFpEF^b^				0.0087
No	5,623 (88.05%)	2,945 (89.08%)	2,678 (86.95%)	
Yes	763 (11.95%)	361 (10.92%)	402 (13.05%)	
Diabetes^b^				< 0.0001
No	5,068 (79.36%)	2,549 (77.1%)	2,519 (81.79%)	
Yes	1,318 (20.64%)	757 (22.9%)	561 (18.21%)	
HTN^b^				0.0343
No	3,637 (56.95%)	1,841 (55.69%)	1,796 (58.31%)	
Yes	2,749 (43.05%)	1,465 (44.31%)	1,284 (41.69%)	
Sex^b^				< 0.0001
Female	2,729 (42.73%)	1,180 (35.69%)	1,549 (50.29%)	
Male	3,657 (57.27%)	2,126 (64.31%)	1,531 (49.71%)	
Ischemic stroke^b^				0.0124
No	6,039 (94.57%)	3,149 (95.25%)	2,890 (93.83%)	
Yes	347 (5.43%)	157 (4.75%)	190 (6.17%)	
Vascular disease^b^				0.0122
No	5,551 (86.92%)	2,840 (85.9%)	2,711 (88.02%)	
Yes	835 (13.08%)	466 (14.1%)	369 (11.98%)	

^a^Data are presented as median (Q1–Q3). ^b^Data are presented as n (%). GFR: glomerular filtration fraction; ORBIT: Outcomes Registry for Better Informed Treatment; HFrEF: heart failure with reduced ejection fraction; HFpEF: heart failure with preserved ejection fraction; HTN: hypertension.

### AC appropriateness and underutilization

The prevalence of AC underutilization and appropriateness is shown in Table 2. Patients in the age group 76–89 had 4.547-fold higher odds (OR = 4.547; 95% confidence interval (CI), 3.827–5.403) to have AC underutilization than those in the age group 50–75. Patients in the age group 76–89 were 108% (OR = 2.077; 95% CI, 1.818–2.374) more likely to have AC appropriateness than those in age group 50–75.

**Table 2:** Prevalence of Underutilization and Appropriateness by Age Groups

	Overall (n = 6,386)	Age 50–75 (n = 3,306)	Age 76–89 (n = 3,080)	P value
Underutilization				< 0.0001
Yes	831 (13.01%)	183 (5.54%)	648 (21.04%)	
No	5,555 (86.99%)	3,123 (94.46%)	2,432 (78.96%)	
Appropriateness				< 0.0001
Yes	1,110 (17.38%)	410 (12.40%)	700 (22.73%)	
No	5,276 (82.62%)	2,896 (87.60%)	2,380 (77.27%)	

Univariate and multivariate analyses were also performed to demonstrate the association of appropriateness and underutilization with CHA_2_DS_2_-VASc score, ORBIT score, glomerular filtration rate (GFR), active bleeding and bleeding (Tables 3–6). History of bleeding (OR = 10.582; 95% CI, 6.467–17.315; P < 0.001) and GFR (OR = 0.985; 95% CI, 0.982–0.988; P < 0.001) were independent predictors of appropriateness. Similar, history of bleeding (OR = 2.402; 95% CI, 1.666–3.462; P < 0.001) and GFR (OR = 1.006; 95% CI, 1.001–1.010; P = 0.0185) were also independent predictors of underutilization.

**Table 3:** Univariate Analysis for the Association of the Appropriateness With CHA_2_DS_2_-VASc, ORBIT Score, GFR, Active Bleeding and Bleeding

Variables	Odds ratio (95% CI)	P value
CHA_2_DS_2_-VASc	1.659 (1.581–1.741)	< 0.0001
ORBIT score	0.665 (0.629–0.703)	< 0.0001
GFR	1.008 (1.005–1.010)	< 0.0001
Active bleeding (reference: yes)	15.902 (8.208–30.809)	< 0.0001
Bleeding (reference: yes)	12.943 (9.584–17.479)	< 0.0001

GFR: glomerular filtration fraction; ORBIT: Outcomes Registry for Better Informed Treatment; CI: confidence interval.

**Table 4:** Multivariate Analysis for the Association of the Appropriateness With CHA_2_DS_2_-VASc, ORBIT Score, GFR, Active Bleeding and Bleeding

Variables	Odds ratio (95% CI)	P value
CHA_2_DS_2_-VASc	1.978 (1.854–2.110)	< 0.0001
ORBIT score	0.723 (0.636–0.821)	< 0.0001
GFR	1.003 (0.999–1.007)	0.1806
Active bleeding (reference: yes)	1.406 (0.620–3.190)	0.4144
Bleeding (reference: yes)	10.582 (6.467–17.315)	< 0.0001

GFR: glomerular filtration fraction; ORBIT: Outcomes Registry for Better Informed Treatment; CI: confidence interval.

**Table 5:** Univariate for the Association of the Underutilization With CHA_2_DS_2_-VASc, ORBIT Score, GFR, Active Bleeding and Bleeding

Variables	Odds ratio (95% CI)	P value
CHA_2_DS_2_-VASc	1.848 (1.749–1.954)	< 0.0001
ORBIT score	4.743 (4.309–5.221)	< 0.0001
GFR	0.985 (0.982–0.988)	< 0.0001
Active bleeding (reference: yes)	6.480 (5.403–7.773)	< 0.0001
Bleeding (reference: yes)	20.431 (16.690–25.011)	< 0.0001

GFR: glomerular filtration fraction; ORBIT: Outcomes Registry for Better Informed Treatment; CI: confidence interval.

**Table 6:** Multivariate Analyses for the Association of the Underutilization With CHA_2_DS_2_-VASc, ORBIT Score, GFR, Active Bleeding and Bleeding

Variables	Odds ratio (95% CI)	P value
CHA_2_DS_2_-VASc	1.993 (1.837–2.164)	< 0.0001
ORBIT score	3.736 (3.163–4.413)	< 0.0001
GFR	1.006 (1.001–1.010)	0.0185
Active bleeding (reference: yes)	1.300 (1.012–1.670)	0.0400
Bleeding (reference: yes)	2.402 (1.666–3.462)	< 0.0001

GFR: glomerular filtration fraction; CI: confidence interval; ORBIT: Outcomes Registry for Better Informed Treatment.

### AC appropriateness and stroke

Comparison of incidence of ischemic stroke according to AC appropriateness was shown in Table 7. Patients with AC appropriateness were 5.8% (OR = 1.058; 95% CI, 0.799–1.401) more likely to have the stroke than patients without appropriateness; however, the results were not statistically significant.

**Table 7:** Prevalence of Appropriateness of Clinical Decisions Against Anticoagulation by Incidence of Ischemic Stroke

	Stroke = yes (n = 347)	Stroke = no (n = 6,039)	P value
Appropriateness			0.6957
Yes	63 (18.16%)	1,047 (17.34%)	
No	284 (81.84%)	4,992 (82.66%)	

## Discussion

In this retrospective single-center study of 6,386 hospitalized patients with AF, we evaluated age-related differences in AC use using CHA_2_DS_2_-VASc for thromboembolic risk and ORBIT for bleeding risk stratification. We observed significantly higher odds of AC underutilization among patients aged 76–89 years compared with those aged 50–75 years, despite elderly patients having higher CHA_2_DS_2_-VASc score and higher stroke risks. These findings highlight the importance of a comprehensive approach balancing the benefits of stroke prevention and risks of bleeding complication in elderly AF patients.

AF prevalence increases markedly with age [7, 8]. In the ATRIA (AnTicoagulation and Risk Factors in Atrial Fibrillation) Registry, among the 17,974 adults with diagnosed AF, 45% were aged 75 years or older. It is projected that the prevalence of AF will increase to more than 5.6 million by 2050, with more than 50% of affected individuals expected to be aged 80 years or older [2]. Large cohort studies have demonstrated that stroke risk rises exponentially in older AF patients, reinforcing the importance of AC therapy [9]. Contemporary guidelines recommend AC therapy for patients with elevated CHA_2_DS_2_-VASc scores, with advanced age incorporated into part of the scoring system [3, 10].

Nonetheless, AC strategy in the elderly patient population presents as a clinical challenge due to the presence of increased risks of stroke, as well as bleeding associated with complex comorbidities and frailty. A meta-analysis that included two randomized controlled trials (NOAH-AFNET 6 and ARTESiA) demonstrated that oral AC reduces risks of ischemic stroke but increases risks of major bleeding. Due to concerns of the risks of bleeding, AC remains frequently underutilized in elderly patients [11]. Even though prior registry analyses, such as the GARFIELD-AF and ORBIT-AF registries, have demonstrated an increasing trend of oral AC use in AF patients, inconsistency and variability remain in those with high risk of stroke, with older age independently associated with both lower prescription rates and higher discontinuation rates [12, 13]. Current data indicate that a more systemic and comprehensive algorithm is needed to address the variability of AC therapy in elderly AF patients.

In our cohort, history of bleeding and reduced GFR were independent predictors of both underutilization and appropriateness of AC therapy. This reflects appropriate clinical caution but also underscores the complexity of balancing thromboembolic and bleeding risks that extend beyond the current available scoring tools. Current guidelines emphasize that bleeding risk assessment should be used to identify modifiable risk factors rather than serve as a reason to withhold AC in patients at high risk of stroke [3, 4, 10]. The results of our study also suggest that further consideration of the type and dosage of AC, especially in patients with chronic kidney disease or end-stage renal disease, is warranted. The study by Shimizu et al [14] showed similar or improved effectiveness of DOACs compared with warfarin in patients with creatinine clearance (CrCl) of 15–30 mL/min, with fewer thromboembolic and death events and less or similar bleeding. Furthermore, the investigators reported that 31.4% of patients with CrCl ≥ 50 mL/min received underdosed DOAC therapy, again emphasizing the frequent issue of DOACs underdosing.

Interestingly, older patients in our study were more likely to meet criteria for “correct” AC classification, suggesting that while underutilization exists, clinicians may be selectively tailoring decisions based on overall risk profiles. This dual observation highlights the importance of individualized care and suggests that age alone does not fully explain prescribing patterns. Structured implementation of risk-based algorithms and shared decision-making frameworks may help reduce inappropriate variation.

### Limitations

This study has several limitations. First, a more detailed classification of AC, including different types, routes of administration for DOACs or low-molecular-weight heparin, and categories of AF (such as existing or new-onset), is needed to further characterize AC use in different age groups and among patients with various types of AF. Given the complexity nature of the included patient population, such as multiple comorbidities, prolonged hospital courses, multiple interventions/procedures, and various socioeconomic/insurance status, a considerable proportion of our patients received more than one type of AC or had limited AC options, thereby complicating the classification of AC in the included patients. Future studies involving more detailed characterization of AC, larger sample sizes, and longer follow-up in the outpatient setting, are required to further explore the nuances of different AC strategies and their influence on clinical outcomes. Second, due to the limitations of retrospective chart review, the exact number of patients in whom AC was withheld, specifically following a major bleed, could not be quantified. Third, future studies using prospective designs and adjusted multivariate models may be better equipped to investigate the independent effect of age (especially among nonagenarians) on AC utilization and to establish causation. Last but not least, this study was not designed as a new-user (incident-user) cohort, as patients already receiving AC prior to the study period were included. Future studies using a new-user design may better evaluate the causal associations between AC initiation and clinical outcomes, as well as explore the differences in clinical outcomes and AC use patterns between new-onset AF and pre-exiting AF.

### Conclusions

Patients in the age group 76–89 were more likely to have AC underutilization than those in the age group 50–75. History of bleeding and GFR were independent predictors of both AC appropriateness and underutilization. These findings highlight the ongoing challenge of balancing stroke prevention and bleeding risk in elderly AF patients and underscore the importance of guideline-directed, individualized AC strategies.

## Data Availability

The data supporting the findings of this study are available from the corresponding author upon reasonable request.
